# Bloodstream infections caused by *Escherichia coli* in onco-haematological patients: Risk factors and mortality in an Italian prospective survey

**DOI:** 10.1371/journal.pone.0224465

**Published:** 2019-10-29

**Authors:** Enrico Maria Trecarichi, Gabriele Giuliano, Chiara Cattaneo, Stelvio Ballanti, Marianna Criscuolo, Anna Candoni, Francesco Marchesi, Marica Laurino, Michelina Dargenio, Rosa Fanci, Mariagiovanna Cefalo, Mario Delia, Angelica Spolzino, Laura Maracci, Gianpaolo Nadali, Alessandro Busca, Maria Ilaria Del Principe, Rosa Daffini, Edoardo Simonetti, Giulia Dragonetti, Maria Elena Zannier, Livio Pagano, Mario Tumbarello, for the Haematologic Malignancies Associated Bloodstream Infections Surveillance (HEMABIS) registry–Sorveglianza Epidemiologica Infezioni Fungine in Emopatie Maligne (SEIFEM) group, Italy

**Affiliations:** 1 Department of Medical and Surgical Sciences, Unit of Infectious and Tropical Diseases, “Magna Graecia” University, Catanzaro, Italy; 2 Istituto di Malattie Infettive, Università Cattolica del Sacro Cuore, Rome, Italy; 3 Hematology, ASST-Spedali Civili, Brescia, Italy; 4 Sezione di Ematologia e Immunologia Clinica, Ospedale Santa Maria della Misericordia, Perugia, Italy; 5 Dipartimento scienze radiologiche radioterapiche ed ematologiche, Fondazione Policlinico Universitario A. Gemelli IRCCS, Rome, Italy; 6 Clinica Ematologica, Centro Trapianti e Terapie Cellulari, Azienda Sanitaria Universitaria Integrata, Udine, Italy; 7 Hematology and Stem Cell Transplant Unit, IRCCS Regina Elena National Cancer Institute, Rome, Italy; 8 Department of Medicine, Haematology Unit, University of Padova, Italy; 9 Unità di Ematologia e Trapianto di cellule staminali, Azienda Ospedaliera Vito Fazzi, Lecce, Italy; 10 SOD complessa di Ematologia, Azienda Ospedaliero-Universitaria Careggi e Università di Firenze, Florence, Italy; 11 Hematology, San Eugenio Hospital, Rome, Italy; 12 Hematology Section, Department of Emergency and Organ Transplant, University of Bari, Bari, Italy; 13 Department of Medicine and Surgery, University of Parma, Parma, Italy; Hematology and BMT Unit, Azienda Ospedaliero-Universitaria di Parma, Parma, Italy; 14 Clinica di Ematologia Azienda Ospedaliero-Universitaria Ospedali Riuniti di Ancona, Ancona, Italy; 15 U.O.C. Ematologia, Azienda Ospedaliera Universitaria Integrata di Verona, Ospedale Borgo Roma, Verona, Italy; 16 Department of Hematology and Stem Cell Transplant Unit, AOU Citta' della Salute e della Scienza, Torino, Italy; 17 Dipartimento di Biomedicina e Prevenzione; Università degli studi di Roma "Tor Vergata", Roma, Italy; 18 Istituto di Ematologia, Università Cattolica del Sacro Cuore, Rome, Italy; 19 Dipartimento di Scienze di Laboratorio e Infettivologiche, Fondazione Policlinico Universitario A. Gemelli IRCCS, Roma, Italy; University of Illinois College of Medicine, UNITED STATES

## Abstract

Bloodstream infections (BSIs) remain life-threatening complications in the clinical course of patients with haematological malignancies (HM) and *Escherichia coli* represent one of the most frequent cause of such infections. In this study, we aimed to describe risk factors for resistance to third generation cephalosporins and prognostic factors, including the impact of third generation cephalosporins resistance, in patients with HM and BSIs caused by *E*. *coli*. Three hundred forty-two cases of *E*. *coli* BSIs were collected during the study period (from January 2016 to December 2017). The percentage of resistance to third generation cephalosporins was 25.7%. In multivariate analysis, the variables recent endoscopic procedures, culture-positive surveillance rectal swabs for multidrug-resistant bacteria, antibiotic prophylaxis with fluoroquinolones, and prolonged neutropenia were independently associated with bloodstream infections caused by a third generation cephalosporins resistant *E*. *coli*. The overall 30-day mortality rate was 7.1%. Cox regression revealed that significant predictors of mortality were acute hepatic failure, septic shock, male sex, refractory/relapsed HM, and third generation cephalosporins resistance by *E*. *coli* isolate. In conclusion, resistance to third generation cephalosporins adversely affected the outcomes of bloodstream infections caused by *E*. *coli* in our cohort of HM patients. We also found a significant correlation between prophylaxis with fluoroquinolones and resistance to third generation cephalosporins by *E*. *coli* isolates.

## Introduction

Although several advances have been made in clinical management of patients with haematological malignancies (HM), bloodstream infections (BSIs) remain life-threatening complications in the clinical course of these patients, with reported crude mortality rate up to 40% [1–6]. A clear shift of bacterial species causing BSI in HM patients has been reported during the last decade from Gram-positives to Gram-negatives, and among the latter, *Enterobacteriaceae*, and in particular *Escherichia coli* (EC), represent the most frequent involved bacterial species [2,6]. Moreover, a worrisome increase in antimicrobial-resistance among *Enterobacteriaceae* has been described in HM patients due mainly to production of extended-spectrum-b-lactamases (ESBLs) and/or carbapenemases by bacterial isolates, which often show a multidrug-resistant (MDR) phenotype with limited treatment options [1–3,6,7]. Resistance to third generation cephalosporins (3GC) by *Enterobacteriaceae*, which represents the major mechanism of antimicrobial resistance among EC isolates, has been reported as independently associated with a poor outcome in HM patients with severe infections caused by EC [4,8,9].

The aim of the present large multicenter study was to identify risk factors for 3GC resistance by EC isolates and prognostic factors, including the impact of 3GC resistance, in HM patients with BSIs caused by EC. The impact of empirical and definitive therapy with carbapenems vs. piperacillin/tazobactam on 30-day mortality was also evaluated.

## Methods

We conducted a prospective cohort study in 15 Italian haematological wards of tertiary care centers or university hospitals participating to Haematologic Malignancies Associated Bloodstream Infections Surveillance (HEMABIS) registry–Sorveglianza Epidemiologica Infezioni Fungine in Emopatie Maligne (SEIFEM) group throughout Italy from January 2016 and December 2017. In participating centers where antimicrobial prophylaxis was administered to high-risk patients, it was performed according to Gruppo Italiano Malattie Ematologiche dell’Adulto (GIMEMA) criteria [10].

All HM patients admitted to participating centers were screened at admission for colonization by multidrug-resistant (MDR) bacterial isolates with a rectal swab; each hospital conducted rectal swab screening according to its own protocols, as described elsewhere [11].

All episodes of BSI caused by EC (EC BSI) occurring in hospitalized HM adult patients were included. Data collected from the hospital charts and the laboratory database included patient demographics, disease and disease stage at time of EC BSI, type of hematopoietic stem cell transplantation (HSCT) (autologous or allogeneic), and outcome of infection; for each bacterial isolate, the antimicrobial susceptibility was recovered and analyzed. All information was entered in case report forms and then recorded in a specific database. Recurrent as well as polymicrobial infections were excluded.

Patients with 3GC resistant (3GCR) EC BSI were compared with those with 3GC susceptible (3GCS) EC BSI in order to identify risk factors for infection by 3GCR EC isolates.

The outcome measured in our EC BSI patients cohort was death within 30 days of the first positive blood culture. Survivor and non-survivor subgroups were compared in order to identify predictors of mortality.

Upon approval of ethic committee of the study coordinating center (Fondazione Policlinico Universitario Agostino Gemelli IRCCS–Università Cattolica del Sacro Cuore), also the ethics committee of each participating site approved the use of the HeMABIS-SEIFEM registry and written informed consent was obtained by each patient.

### Definitions

The following terms were defined prior to data analysis:

EC BSI was defined as an infection manifested by the presence in at least 1 blood culture that grew an EC strain.

Neutropenia was defined as an absolute neutrophil count (ANC) <500 neutrophils/μL at the onset of BSI; neutropenia was considered prolonged if the duration was ≥10 days and severe if ANC was <100 neutrophils/μL.

EC BSI was considered hospital-acquired if the index culture had been collected >48 hours after admission and signs and symptoms of infection were absent at admission. If cultures had been collected ≤48 hours after the admission date, the isolate was classified as healthcare-associated or community-acquired [12].

The empirical therapy was classified as *inadequate* if antibiogram demonstrated resistance of EC isolate to the administered antimicrobial(s).

Septic shock was defined according to Surviving Sepsis Campaign criteria [13].

### Statistical analysis

Continuous variables were compared by Student’s *t* test for normally distributed variables and the Mann-Whitney U test for non-normally distributed variables. Categorical variables were evaluated using the χ^2^ or two-tailed Fisher's exact test. Odds ratios (ORs) and 95% confidence intervals (CIs) were calculated to evaluate the strength of any association that emerged. Values are expressed as means ± standard deviation (SD) (continuous variables), or as percentages of the group from which they were derived (categorical variables). Two-tailed tests were used to determine statistical significance; a P value of <0.05 was considered significant. Multivariate analysis was used to identify independent risk factors for 3GCR EC BSI and Cox regression analysis was conducted to identify independent risk factors for 30-day mortality. Variables emerging from univariate analyses for 3GCR EC BSI and 30-day mortality with P values of <0.1 were included in a backward stepwise manner in the multivariate and the Cox regression models, respectively,. The Kaplan-Meier method was used for survival analysis. All statistical analyses were performed using the Intercooled Stata program, version 11, for Windows (Stata Corporation, College Station, Texas, USA).

## Results

**Fig 1:**
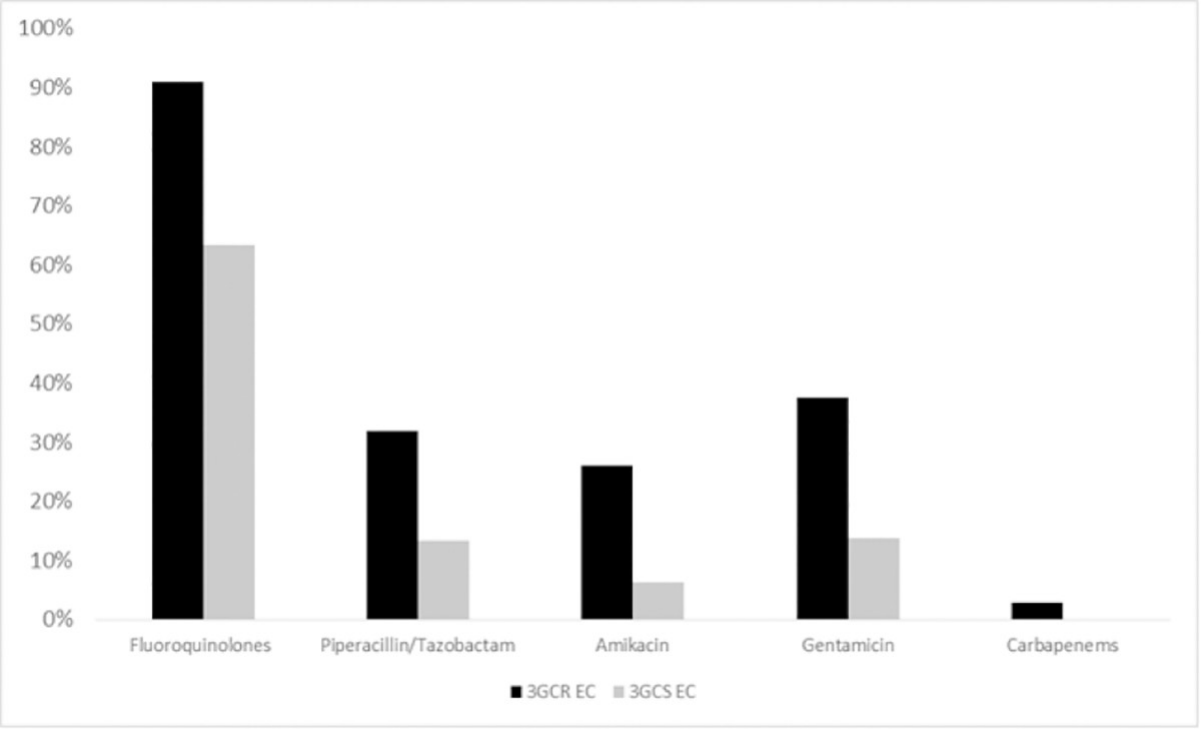
Percentages of resistance to the more commonly used antibiotics for treatment of *Escherichia coli* infections according to resistance to 3^rd^ generation cephalosporins.

A total of 342 cases of EC BSI were collected during the study period. The rate of resistance to 3GC among EC isolates was 25.7% (88/342). Compared to 3GCS EC isolates, 3GCR EC isolates were more likely to be resistant to fluoroquinolones (FQ) (80/88, 90.9%, vs. 161/254, 63.4%; P<0.001), piperacillin/tazobactam (25/88, 28.4%, vs. 34/254, 13.4%; P<0.001), amikacin (23/88, 26.1%, vs. 16/254, 6.3%; P<0.001), and gentamicin (33/88, 37.5%, vs. 35/254, 13.8%; P<0.001) (**Fig 1**). Only two EC isolates (0.6%), both resistant to 3GC, displayed resistance to carbapenems.

### Risk factors for third-generation resistance in patients with EC BSI

In **Table 1** clinical and epidemiological characteristics of EC BSI cohort patients according to 3GC resistance are shown. Compared to patients with 3GCS EC BSI, those with 3GCR EC BSI more likely had undergone endoscopic procedures (9/88, 10.2%, vs. 7/254, 2.8%; P = 0.004) and/or parenteral nutrition (25/88, 28.4%, vs. 44/254, 17.3%; P = 0.02) and/or allogeneic-matched unrelated HSCT (9/88, 10.2%, vs. 9/254, 3.5%; P = 0.01), had prolonged neutropenia (53/88, 60.2%, vs. 118/254, 46.5%; P = 0.02), had previous MDR Gram-negative bacteria culture-positive surveillance rectal swabs (32/88, 36.4%, vs. 42/254, 16.5%; P<0.001), and received antibiotic prophylaxis with FQ (56/88, 63.6%, vs. 119/254, 46.9%; P = 0.006). Patients suffering from multiple myeloma had more frequently a 3GCS EC BSI (26/254, 10.2% vs. 3/88, 3.4%; P = 0.05). No other variable was associated with 3GCS EC BSI, with particular reference to demographic characteristics, type of HM or primary site of BSI.

**Table 1:** Clinical and demographic characteristics of patients with hematological malignancies and BSI caused by *Escherichia coli* according to resistance of isolate to third-generation cephalosporins.

Variables	3GCR EC BSI (n = 88)	3GCS EC BSI (n = 254)	P values
*Demographic information*			
Male sex	49 (55.7)	149 (58.7)	0.62
Age > 65 years	18 (20.5)	61 (24.0)	0.49
*Patient’s characteristics*			
Indwelling CVC	81 (92.0)	223 (87.8)	0.27
Indwelling urinary catheter	13 (14.8)	40 (15.7)	0.82
Endoscopic procedures^§^	9 (10.2)	7 (2.8)	**0.004**
Parenteral nutrition^§^	25 (28.4)	44 (17.3)	**0.02**
Diabetes mellitus	11 (12.5)	32 (12.6)	0.98
Chronic hepatic failure	1 (1.1)	16 (6.3)	0.30
Chronic renal failure	1 (1.1)	13 (5.1)	0.10
PMN < 500/mmc	80 (90.9)	227 (89.3)	0.68
PMN < 100/mmc	77 (87.5)	206 (81.1)	0.17
PMN < 500/mmc for at least 10 days	54 (61.4)	118 (46.5)	**0.02**
Corticosteroid treatment^§^	33 (37.5)	119 (46.8)	0.12
Chemotherapy^§^	69 (78.4)	210 (82.7)	0.37
Radiotherapy^§^	3 (3.4)	17 (6.7)	0.25
Therapy with monoclonal antibodies^§^	14 (15.9)	41 (16.1)	0.95
Previous antibiotic therapy^^^	28 (31.8)	52 (20.5)	**0.03**
*Hematological malignancy*			
Acute myeloid leukemia/myelodysplastic syndrome	51 (58.0)	132 (52.0)	0.33
Chronic myeloid leukemia	2 (2.3)	2 (0.8)	0.26
Acute lymphoblastic leukemia	8 (9.1)	24 (9.4)	0.92
Lymphomas/chronic lymphoid leukemia	24 (27.3)	70 (27.6)	0.95
Multiple Myeloma	3 (3.4)	26 (10.2)	**0.05**
*Stage of Hematological Disease*			
Newly diagnosed / Relapsed after 1st remission	21 (23.7)	50 (19.7)	0.40
Complete remission	17 (19.3)	49 (19.3)	0.99
Refractory / Relapsed after 2 or more remissions	17 (19.3)	70 (27.6)	0.12
Hematopoietic stem cell transplantation	33 (37.5)	85 (33.5)	0.49
Autologous	16 (18.2)	50 (19.7)	0.75
Allogeneic-Matched Related	3 (3.4)	11 (4.3)	0.70
Allogeneic-Matched Unrelated	9 (10.2)	9 (3.5)	**0.01**
Allogeneic-Mismatched	5 (5.7)	15 (5.9)	0.94
*Characteristics of BBSI*			
Hospital acquired	72 (81.8)	216 (85.1)	0.47
Healthcare-associated	16 (18.2)	38 (15.0)	0.47
*Primary site of BSI*			
Urinary tract	3 (3.4)	13 (5.1)	0.51
Respiratory tract	2 (2.3)	5 (2.0)	0.86
CVC	25 (28.4)	61 (24.0)	0.41
Others	3 (3.4)	12 (4.7)	0.60
Unidentified	58 (65.9)	167 (65.7)	0.97
MDR bacteria culture-positive surveillance rectal swabs^§^	32 (36.4)	42 (16.5)	**<0.001**
Antibiotic fluoroquinolones prophylaxis	56 (63.6)	119 (46.9)	**0.006**
Antifungal prophylaxis	49 (55.7)	137 (53.9)	0.77
Time at risk (days, mean ± SD)	15 ± 14	13 ± 9	0.22
Inadequate initial antimicrobial therapy	25 (28.4)	26 (10.2)	**<0.001**
21-day mortality	12 (13.6)	12 (4.7)	**0.004**

Abbreviations: CVC, central venous catheter; PICC, peripherally inserted central catheter; PMN, polymorphonuclear leukocytes; 3GCR, 3^rd^ generation cephalosporins resistant; 3GCS, 3^rd^ generation cephalosporins susceptible.

^§^During the last 30 days

^ During the last 90 days

In multivariate analysis, the variables recent endoscopic procedures (OR 3.68, 95% CI 1.23–11.01; P = 0.02), MDR bacteria culture-positive surveillance rectal swabs (OR 2.81, 95% CI 1.59–4.95; P<0.001), antibiotic prophylaxis with FQ (OR 1.94, 95% CI 1.16–3.28; P = 0.01), and prolonged neutropenia (OR 1.82, 95% CI 1.08–3.06; P = 0.02) were found to be independently associated with BSI caused by a 3GCR EC isolate (**Table 2**).

**Table 2:** Multivariate analysis of risk factors for 3^rd^ generation cephaloporins in patients with hematological malignancies and BSI caused by *Escherichia coli*.

Variables	OR	(95% IC)	P values
Recent endoscopic procedures	3.68	(1.23–11.04)	0.02
MDR bacteria culture-positive surveillance rectal swabs	2.81	(1.59–4.95)	<0.001
Antibiotic prophylaxis with fluoroquinolones	1.95	(1.16–3.28)	0.01
PMN < 500/mmc for at least 10 days	1.82	(1.08–3.06)	0.02

### Risk factors for 30-day mortality in patients with EC BSI

**Fig 2:**
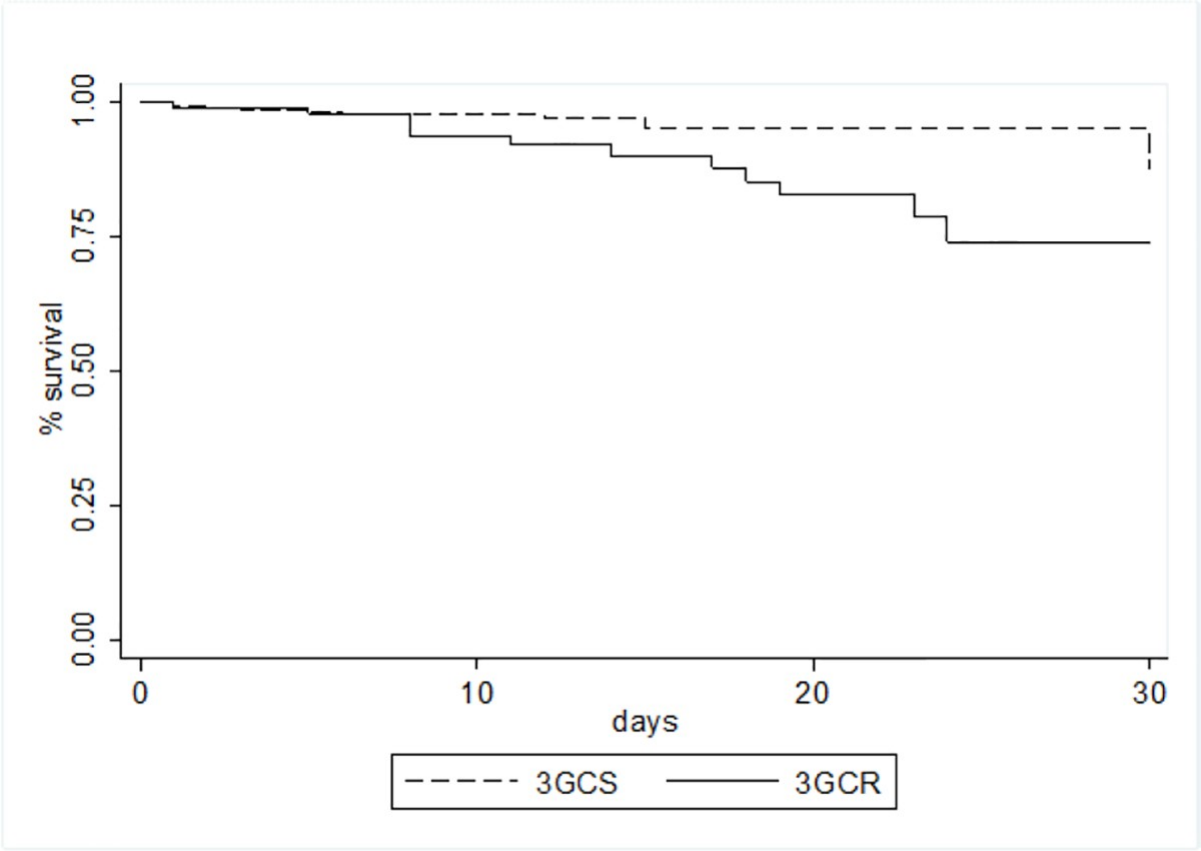
Kaplan-Meier survival estimates among patients with bloodstream infections caused by 3^rd^ generation cephalosporins resistant and 3^rd^ generation cephalosporins susceptible *Escherichia coli*.

The 24 (7.1%) patients who died within 30 days of EC BSI onset included 12 of the 88 (13.6%) whose EC isolate was resistant to 3GC and 12/254 (4.7%) who had a BSI caused by a 3GCS EC (P = 0.004). The 30-day survival distributions were also significantly different in HM patients with 3GCR EC BSI vs. 3GCS EC BSI (P = 0.007)(**Fig 2**).

In univariate analysis (described in **Table 3**), variables associated to 30-day mortality were: septic shock (14/24, 58.3%, vs. 46/318, 14.5%; P<0.001), altered state of consciousness (11/24, 45.8%, vs. 23/318, 7.2%; P<0.001), acute renal (10/24, 41.7%, vs. 18/318, 5.6%; P<0.001) and/or respiratory (8/24, 33.3%, vs. 11/318, 3.4%; P<0.001) and/or hepatic failure (5/24, 20.8%, vs. 1/318, 0.3%; P<0.001), and 3GC resistance by EC isolate(12/24, 50%, vs. 76/318, 23.9%; P = 0.004). On the other hand, autologous HSCT (1/24, 4.2%, vs. 65/318, 20.4%; P = 0.05) and/or complete remission stage of HM disease (0/24 vs. 66/318, 20.8%; P = 0.01) were significantly associated with survival. No other variable was associate to 30-day mortality, in particular with regard to patient’s characteristics (including neutropenia, regardless of severity and duration), type of HM, and inadequate initial antimicrobial therapy.

**Table 3:** Univariate analysis of risk factors for mortality in patients with hematological malignancies and BSI caused by *Escherichia coli*.

Variables	Non-survivors (n = 24)	Survivors (n = 318)	OR (95% IC)	P values
*Demographic information*				
Male sex	19 (79.2)	179 (56.3)	2.95 (1.03–10.33)	**0.02**
Age > 65 years	8 (33.3)	71 (22.3)	1.73 (0.61–4.51)	0.21
*Patient’s characteristics*				
Diabetes mellitus	5 (20.8)	38 (11.9)	1.93 (0.53–5.78)	0.20
Chronic hepatic failure	3 (12.5)	16 (5.0)	2.69 (0.46–10.522)	0.12
Chronic renal failure	2 (8.3)	12 (3.8)	2.31 (0.23–11.42)	0.27
PMN < 500/mmc	20 (83.3)	287 (90.3)	0.54 (0.16–2.31)	0.28
PMN < 100/mmc	17 (70.8)	266 (83.6)	0.47 (0.17–1.42)	0.10
PMN < 500/mmc for at least 10 days	9 (37.5)	163 (51.3)	0.57 (0.21–1.43)	0.19
Corticosteroid treatment	11 (45.8)	141 (44.3)	1.06 (0.41–2.65)	0.88
Chemotherapy	16 (66.7)	263 (82.7)	0.41 (0.15–1.19)	0.43
Radiotherapy	1 (4.2)	19 (6.0)	0.68 (0.01–4.71)	0.71
Therapy with monoclonal antibodies	5 (20.8)	50 (15.7)	1.41 (0.39–4.14)	0.51
*Hematological malignancy*				
Acute myeloid leukemia/ myelodysplastic syndrome	11 (45.8)	172 (54.1)	0.71 (0.28–1.79)	0.43
Chronic myeloid leukemia	0	4 (1.3)	-	0.58
Acute lymphoblastic leukemia	3 (12.5)	29 (9.1)	1.42 (0.25–5.20)	0.58
Lymphomas/chronic lymphoid leukemia	8 (33.3)	86 (27.1)	1.34 (0.48–3.48)	0.50
Multiple Myeloma	2 (8.3)	27 (8.5)	0.97 (0.10–4.35)	0.97
*Stage of Hematological Disease*				
Newly diagnosed / Relapsed after 1st remission	7 (29.2)	64 (20.1)	1.63 (0.54–4.35)	0.29
Complete remission	0	66 (20.8)	-	**0.01**
Refractory / Relapsed after 2 or more remissions	10 (41.7)	77 (24.2)	2.23 (0.84–5.64)	0.06
*Hematopoietic stem cell transplantation*	7 (29.2)	111 (34.9)	0.76 (0.26–2.02)	0.56
Autologous	1 (4.2)	65 (20.4)	0.16 (0.01–1.08)	**0.05**
Allogeneic-Matched Related	2 (8.3)	12 (3.8)	2.31 (0.23–11.42)	0.27
Allogeneic-Matched Unrelated	1 (4.2)	17 (5.3)	0.76 (0.01–5.37)	0.80
Allogeneic-Mismatched	3 (12.5)	17 (5.3)	2.52 (0.43–9.78)	0.15
*Clinical characteristics of BSI*				
Septic shock	14 (58.3)	46 (14.5)	8.27 (3.17–21.99)	**<0.001**
Altered state of consciousness	11 (45.8)	23 (7.2)	10.85 (3.88–29.34)	**<0.001**
Acute renal failure	10 (41.7)	18 (5.7)	11.90 (4.06–33.26)	**<0.001**
Acute respiratory failure	8 (33.3)	11 (3.5)	13.95 (4.18–43.85)	**<0.001**
Acute hepatic failure	5 (20.8)	1 (0.3)	83.42 (8.39–3955.44)	**<0.001**
Granulocyte transfusion	1 (4.2)	11 (3.5)	1.21 (0.02–9.05)	0.58
Antibiotic fluoroquinolones prophylaxis	12 (50.0)	163 (51.3)	0.95 (0.37–2.39)	0.90
Inadequate initial antimicrobial therapy	2 (8.3)	49 (15.4)	0.49 (0.05–2.14)	0.34
3^rd^ generation cephalosporins resistance by EC isolate	12 (50.0)	76 (23.9)	3.18 (1.24–8.04)	**0.004**

Abbreviations: PMN, polymorphonuclear leukocytes.

In Cox regression, significant predictors of mortality were acute hepatic failure (HR 9.90, 95% CI 3.08–31.73; P<0.001), septic shock (HR 8.56, 95% CI 3.33–21.95; P<0.001), male sex (HR 6.46, 95% CI 2.19–19.01; P = 0.001), refractory/relapsed HM (HR 3.25, 95% CI 1.35–7.83; P = 0.008), and 3GC resistance by EC isolate (HR 3.18, 95% CI 1.36–7.43; P = 0.007) (**Table 4**).

**Table 4:** Cox regression analysis for mortality in patients with hematological malignancies and BSI caused by *Escherichia coli*.

Variables	HR	(95% IC)	P values
Acute hepatic failure	9.90	(3.08–31.73)	<0.001
Septic shock	8.56	(3.33–21.95)	<0.001
Male sex	6.46	(2.19–19.01)	0.001
Refractory/Relapsed HM	3.25	(1.35–7.83)	0.008
3^rd^ generation cephalosporins resistance by EC isolate	3.18	(1.36–7.43)	0.007

### Empirical and definitive antibiotic treatment in patients with EC BSI

Empirical antimicrobial treatment was started within a few hours after the index blood culture was drawn in all 342 cases. In vitro susceptibility testing results showed that empirical therapy was inadequate in 51/342 (14.9%) cases. The rate of initial inadequate antibiotic therapy was significantly more frequent in patients with 3GCR EC BSI compared to those with 3GCS EC BSI (25/88, 28.4% vs. 26/254, 10.2%; P<0.001).

Among the 88 patients suffering from 3GCR EC BSI, the 30-day mortality rate did not differ significantly between those initially treated with an antibiotic regimen containing piperacillin/tazobactam compared to other regimens (7/42, 16.7% vs. 5/46, 10.9%; P = 0.42), as well as there was not any significant difference in mortality between patients who had empirically received an antibiotic regimen including a carbapenem compared those treated with other regimens (3/31, 9.7% vs 9/57, 15.8%; P = 0.42). Including only patients who had received empirical regimens based on piperacillin/tazobactam or carbapenems (73/88), there was not any significant difference between those treated with carbapenems compared to those treated with piperacillin/tazobactam (3/31, 9.7% vs. 7/42, 16.7%; P = 0.39).

Similarly, among the 84/88 patients with carbapenem-susceptible EC BSI and who had been treated with definitive regimens based on carbapenems or piperacillin/tazobactam, there were no significant differences in the 30-day mortality rates between those treated with carbapenems vs. those treated with piperacillin/tazobactam (9/58, 15.5% vs. 2/26, 7.7%; P = 0.32).

## Discussion

In the present study conducted in 15 Italian Haematological Units we found that the rate of resistance to 3GC among EC isolates was 25.7% (88/342). Compared to previous epidemiological data from the HeMABIS-SEIFEM registry collected from 2009 to 2012, the percentage of resistance to 3GC among EC isolates has not increased, rather it is slightly reduced (25.7% vs. 30%) [2]. Similarly, resistance to 3GC among EC isolates was slightly lower than that reported in Italian general population during the same period (2016 to 2017) by Antimicrobial Resistance Surveillance in Europe (EARSS) (25.7% vs. 29.6%) [14] and it was substantially in line with reports conducted in HM or cancer patients in other countries [9,15–17]. These data indicate that, in contrast to other MDR Gram-negative bacteria (e.g. carbapenem-resistant *Klebsiella pneumoniae*) for which a worrisome increasing trend in frequency has been recently highlighted in the HeMABIS-SEIFEM registry previous survey in HM patients [3], the rate of resistance to 3GC among EC isolates remained stable during the last years. Interestingly, the result was considerably decreased compared to what reported in studies published worldwide in cancer patients from 2007 to 2013 (mean 53.3% rate of resistance to ceftazidime) [1].

We investigated risk factors for 3GCR EC BSI and found that recent endoscopic procedures, previous culture-positive surveillance rectal swabs, prolonged neutropenia, and antibiotic prophylaxis with FQ were independently associated with 3GCR EC BSI. While the first three risk factors have been widely described as related with BSI due to ESBL-producing or MDR *Enterobacteriaceae* both in general population and in onco-haematological patients [11,18–20], the role of FQ prophylaxis in increased rate of infections caused by MDR Gram-negative bacteria remains actually a topic widely debated, also considering the burden of antimicrobial resistance, particularly in *Enterobacteriaceae*, worldwide throughout the last decades [1,21]. In line with our results, previous exposure to antimicrobials, and in particular FQ, has been largely demonstrated as one of the most important risk factors for colonization and/or infection by ESBL- or carbapenemase-producing *Enterobacteriaceae* both in general population and HM patients [1,4,9,16–18,22]. To this regard, European Conference on Infections in Leukemia (ECIL) has recently conducted a meta-analysis in order to evaluate the role of FQ prophylaxis in onco-haematological patients; it concluded that FQ prophylaxis did not have effect on mortality while was associated with lower rate of BSI and episodes of febrile neutropenia without a clear effect of the background rate of FQ resistance on the efficacy of FQ prophylaxis [23]. Despite FQ prophylaxis independently predicted 3GCR, no correlation was found between FQ prophylaxis and 30-day mortality in our cohort of HM patients, and this could at least be partially due to the specific focus of the present study on EC BSIs.

The overall 30-day mortality rate in our cohort was 7.0% (24/342); compared to previous studies on EC BSI in HM or cancer patients, it was considerably lower [4,7–9].

By cox regression analysis, the most important risk factors for mortality in our cohort were acute respiratory failure and septic shock at EC BSI onset; not surprisingly, similar variables (e.g. admission to Intensive Care Unit, etc.), indicating clinical severity of BSI, had been reported to be associated with mortality in several previous studies conducted on HM or cancer patients with EC BSI [4,7,9]. Male sex and refractory/relapsed HM also were found to be independently associated with mortality, as previously reported in general or HM populations suffering from bacterial BSIs [24,25]. Finally, the resistance to 3GC by EC isolate emerged as variable independently associated with 30-day mortality in our cohort. In line with our data, antibiotic-resistance by Gram-negative bacteria has been reported to be strongly associated with mortality in several previous studies conducted in HM patients with BSI [1,26]. In particular, ESBL production (i.e. resistance to 3GC) has been associated with mortality in HM or cancer patients with EC BSI [4,8,9]. The relationship between infection with antibiotic resistant bacteria and mortality is not completely clear and it has been often related to a delay in the initiation of an appropriate antibiotic therapy [1,8]. Of note, in our study, inadequacy of empirical antibiotic therapy, although significantly more frequent in patients with 3GCR EC BSI, was not associated with a poorer outcome, in line with other studies [7,9,15,16].

A recent randomized clinical trial investigating the effect of definitive antimicrobial therapy with piperacillin-tazobactam vs. meropenem on 30-day mortality in patients with BSIs caused by ceftriaxone-resistant EC or *Klebsiella pneumoniae* isolates, demonstrated that definitive treatment with piperacillin-tazobactam failed to be non-inferior to meropenem, thus not supporting the use of piperacillin-tazobactam in this setting [27]. In our prospective observational cohort of 88 3GCR EC BSI patients, we did not find any significant difference in 30-day mortality between those treated both empirically or definitively with regimens based on piperacillin-tazobactam vs. meropenem. These findings are in line with those of Gudiol et al. who have recently conducted a study comparing the efficacy of β-Lactam/β-lactamase inhibitors (BLBLIs) to carbapenems in two cohorts (empirical therapy cohort, 174 patients, and definitive therapy cohort, 251 patients) of haematological neutropenic patients with ESBL-producing *Enterobacteriaceae* BSI, and did not find any significant difference in 30-day case fatality rates [28]. Probably the role of BLBLIs, and in particular of piperacillin-tazobactam, in treatment 3GCR EC infections should be further investigated, at least in settings as those of HM.

This study has some limitations. As it was carried out in a country with a high prevalence of 3GCR EC, the results may not be generalized, in particular for countries with a different epidemiological context. In addition, the mechanisms underlying resistance to 3GC in EC isolates have not been investigated.

In conclusion, in this large multicenter Italian prospective study, we found that, although the rate of 3GCR EC remained stable trough the recent years, resistance to 3GC adversely affected the outcomes of EC BSI in HM patients. We also found a significant correlation between FQ prophylaxis and resistance to 3GC by EC isolates, whereas piperacillin/tazobactam as empirical therapy in the overall cohort of EC BSI patients and as etiological therapy among 3GCR EC BSI patients was not associated with a worse outcome compared to carbapenem-based antimicrobial regimens. Further studies are necessary to investigate the actual role of FQ prophylaxis in HM patients, especially in settings with high level of antibiotic resistance, and possible role of piperacillin/tazobactam in treatment of EC BSIs in HM patients.
